# COVID-19 Disease Burden Related to Social Vulnerability and Comorbidities: Challenges to Tuberculosis Control

**DOI:** 10.3390/ijerph19063597

**Published:** 2022-03-18

**Authors:** Yeo Wool Lee, Jeong Yeon Seon, Seung Heon Lee, In Hwan Oh

**Affiliations:** 1 Department of Preventive Medicine, Korea University College of Medicine, Seoul 02841, Korea; skdmldudn@korea.ac.kr; 2 Department of Preventive Medicine, Kyung Hee University College of Medicine, Seoul 02447, Korea; jeongyeon210@naver.com; 3 Division of Pulmonary, Sleep and Critical Care Medicine, Department of Internal Medicine, Korea University Ansan Hospital, Ansan-si 15355, Korea

**Keywords:** COVID-19 pandemic, tuberculosis patient, severity of illness, preventive intervention, vulnerable group

## Abstract

Purpose: The first coronavirus disease (COVID-19) spike and subsequent pandemic in South Korea were rapid and disruptive. Government response measures for disadvantaged groups against infectious disease should be prioritized based on evidence and affordability. We investigated whether COVID-19 infection, intensive care unit (ICU) care, and mortality from COVID-19 are related to social and medical vulnerability, including tuberculosis (TB). Patients and Methods: Using the National Health Insurance Service COVID-19 database in South Korea, we analyzed 129,128 patients, including controls, from 1 January to 30 May 2020, during the early stage of the COVID-19 epidemic. The relationship between health insurance premiums (representing socioeconomic status), the Charlson comorbidity index (CCI) score for the severity of the underlying disease, and additional TB diagnosis was analyzed using the chi-square test and logistic regression. Results: For the demographics, 3244 out of 51,783 men (6.3%) and 4836 out of 77,345 women (6.3%) were infected with Severe Acute Respiratory Syndrome Coronavirus 2 (SARS-CoV-2). COVID-19 infection, ICU care, and mortality were related to older age (*p* < 0.001) and lower health insurance premium levels (*p* < 0.05). Regarding the CCI score, the CCI score, COVID-19 infection, and mortality increased (*p* < 0.0001). In terms of premium level, the highest group showed a lower risk of infection (OR 0.52, 0.48-0.57, *p* = 0.004), ICU care (OR 0.59, 0.46-0.75, *p* < 0.001), and mortality (OR 0.51, 0.32-0.78, *p* = 0.016) than the medical aid group. TB was related to ICU care for COVID-19 (OR 4.27, 1.27-14.38, *p* = 0.018). Conclusion: In the early epidemic, SARS-CoV-2 infection, ICU admission, and mortality from COVID-19 increased in socioeconomically and physically vulnerable groups. However, the relationship between tuberculosis, COVID-19 and mortality was not definite because of the possible under-reporting of TB cases and the relatively small number of TB patients.

## 1. Introduction

The World Health Organization (WHO) declared the outbreak of the novel coronavirus disease (COVID-19) as a public health emergency of international concern on 30 January 2020, and recorded 209,876,613 cases and 4,400,284 deaths from COVID-19 by August 19 [1]. South Korea was one of the countries affected by COVID-19 in the initial epidemic; the first confirmed case was reported on 20 January 2020. As of 19 August 2021, there had been 232,859 confirmed cases of COVID-19 in South Korea, resulting in 2197 deaths (see the WHO COVID-19 Dashboard).

In many infectious diseases, including COVID-19, hygiene and environmental control are very important in order to block the infection connection chain, especially in socially and medically vulnerable groups [2]. Even though government response policies for disadvantaged groups should have been prioritized based on evidence and affordability, the initial COVID-19 surge and consecutive pandemic were abrupt and disruptive to the earlier-affected Asian countries [3]. Socially vulnerable groups such as intellectually disabled people were expected to be affected more seriously in the initial stage of the COVID-19 pandemic [4].

A consistent finding in this pandemic is that patients with comorbidities are more likely to be afflicted by the virus. A meta-analysis of eight studies and 46,248 patients with Severe Acute Respiratory Syndrome Coronavirus 2 (SARS-CoV-2) infection confirmed by molecular diagnostics showed that comorbidities were powerful predictors of the need for hospitalization and poor outcomes [5]. The suggested comorbidities are diabetes, cardiac disease, chronic renal disease, bronchiectasis, and obesity [6]. Given that most fatal cases develop from respiratory failure, such as adult respiratory distress syndrome (ARDS) requiring ventilator care or extracorporeal membrane oxygenation (ECMO), pre-existing or co-infected respiratory disease could critically influence the clinical course of COVID-19 [2].

Individuals with latent or active tuberculosis (TB) were more susceptible to SARS-CoV-2 infection, according to an observational study from Wuhan, the pandemic’s epicenter [7]. Infection with *Mycobacterium tuberculosis* was found to be a more common comorbidity in COVID-19 (36%) than diabetes (25%), hypertension (22%), ischemic heart disease (8%), and chronic obstructive lung disease and chronic obstructive pulmonary disease (COPD) (6%) in China. *M. tuberculosis* co-infection was also associated with more severe COVID-19 and more rapid progression. Therefore, considering the intermediate TB burden in the Republic of Korea (hereafter Korea), where 19,933 new TB cases (38.8 per 100,000 population) and 1600 TB-related deaths were reported in 2020 [8], the possible links between TB and COVID-19 should be clarified [9].

We aimed to investigate the ways in which infection with COVID-19, ICU care, and mortality from COVID-19 were related to social and medical vulnerabilities, as well as TB.

## 2. Material and Methods

### 2.1. Data Collection and Study Participants

**Figure 1:**
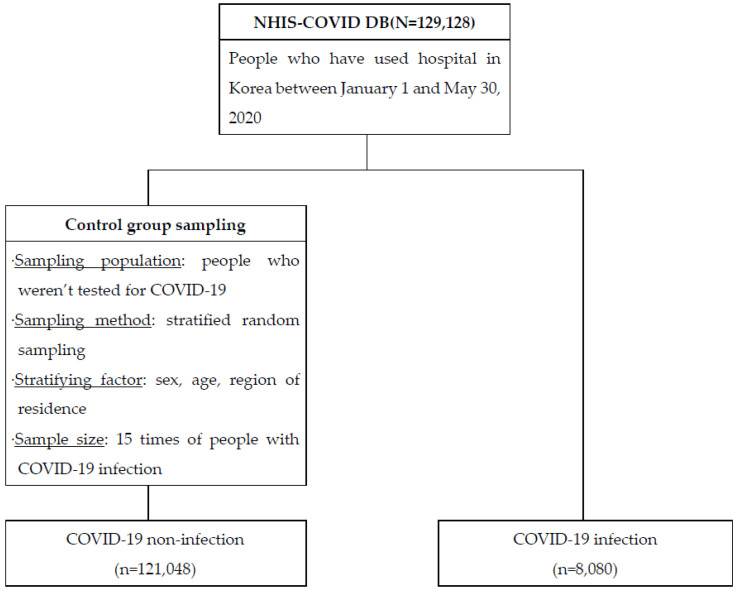
Flow chart for people included in the analysis.

The purpose of this study was to determine the severity of SARS-CoV-2 infection in the tuberculosis population compared to the total population, in order to evaluate the appropriateness of the recent government directives, by analyzing SARS-CoV-2 infection, intensive care unit (ICU) admission, and COVID-19 death rates. In Korea’s health insurance system, public insurance is operated and funded by a single insurer, the National Health Insurance Service (NHIS). In Korea, all patients with confirmed COVID-19 are hospitalized. Therefore, all medical claims, including those for COVID-19 patients, are collected from the NHIS. We used the NHIS-COVID database (DB) for this analysis, which is given by the NHIS to facilitate research investigations that are based on the evidence for coronavirus therapy and policy implementation. This national data includes one’s insurance status, insurance premiums, and medical examination results. The data source includes all medical records from January 2015 to July 2020 for COVID-19 patients and controls from January 1 to 30 May 2020. The control group was a stratified sample of patients without a history of COVID-19 testing on a scale 15 times that of the COVID-19 patients. Stratification factors including sex (male, female), age group (0–9, 10–19, 20–29, 30–39, 40–49, 50–59, 60–69, 70–79, 80+), and residence (Seoul, Daegu, Gyeonggi-do, Gyeongsangbuk-do, Others) were considered based on their high prevalence, and random samples were drawn within the same groups. In this study, 129,128 patients included in the NHIS-COVID DB were analyzed (Figure 1), of which 121,048 were not infected and 8080 were infected with SARS-CoV-2, and the association between TB and SARS-CoV-2 infection was analyzed, in addition to the effect of the presence of tuberculosis on ICU admission and mortality in COVID-19 patients.

### 2.2. Statistical Analysis

A chi-square test was performed to confirm the association between TB diagnosis, SARS-CoV-2 infection, ICU admission, and COVID-19 death, and to examine the characteristics of patients that affected the outcome variables (Table 1). The variables reviewed for analysis were gender, age, residence, health insurance premium, Charlson comorbidity index (CCI) score, degree of disability, type of disability, disability, and tuberculosis diagnosis. The ages were categorized as 0–59, 60–69, 70–79, and 80+ years, and residence was classified based on regions with many COVID-19 confirmed cases (Seoul, Daegu, Gyeonggi-do, Gyeongsangbuk-do, and Others). The health insurance premium was used as a variable to reflect socioeconomic status, a main variable of interest in this study. In Korea, health insurance premiums are paid according to income level. Medical aid recipients fall under the low-income class, and do not pay health insurance premiums; those who do pay health insurance premiums are classified into the 1st quantile (lowest), 2nd quantile, 3rd quantile, 4th quantile, and 5th quantile (highest) according to the amount paid. The CCI score refers to the severity of the underlying disease, and the updated weights suggested by Quan et al. were assigned based on the 2019 medical records [10]. The characteristics of the people with disabilities were classified according to the degree of disability and type of disability. TB diagnosis was based on the medical record in 2019, and a history of TB as the main diagnosis or TB-related special calculation codes (‘R7860’, ‘V206’, ‘V246’, ‘V000’, ‘F009’, ‘F010’) were defined as a patient with TB. The ICD-10 codes for tuberculosis diagnosis were defined as ‘A15’, ‘A16’, ‘A17’, ‘A19’, ‘P370’, ‘U843’, and ‘U880’.

Logistic regression analysis was performed in order to analyze the effect of TB diagnosis on SARS-CoV-2 infection, ICU admission, and COVID-19 death. In the logistic regression model in which SARS-CoV-2 infection was set as an outcome variable, health insurance premiums, CCI scores, and disability were used as independent and adjustment variables. In the model that considered ICU admission and COVID-19 mortality due to SARS-CoV-2 infection as outcome variables, gender, age, region, CCI score, and disability were input as independent variables. The statistical analysis was performed using SAS 9.4, and statistical significance was set at *p* < 0.05.

### 2.3. Ethics Statement

This study followed Korean rules for de-identification of personal data and was accepted as a review-exempt study by Kyung Hee University’s Institutional Review Board (IRB No. KHSIRB-20-301(EA)). The board waived informed consent because the study used de-identified data.

## 3. Results

### 3.1. COVID-19 Patient Characteristics

The demographic characteristics of 8080 individuals with SARS-CoV-2 infections, 1020 ICU admissions, and 248 COVID-19 deaths are shown in Table 1. A Chi-square test was performed to compare the rates of SARS-CoV-2 infection, ICU admission, and COVID-19 death with the characteristics of the COVID-19 patients, and to determine the statistical significance. Looking at SARS-CoV-2 infections by demographic characteristics, 3244 out of 51,783 men (6.3%) and 4836 out of 77,345 women (6.3%) were infected with SARS-CoV-2. The infection rates according to health insurance premium levels were the highest for patients with medical benefits (10.9%). The higher the CCI score, the higher the SARS-CoV-2 infection rate (*p* < 0.001).

Approximately 12.6% of the patients were admitted to the ICU, and 3.1% of the patients died (*p* < 0.001). The intensive care unit admission and death rates were higher in male (15.9% and 4.2%, respectively) than in female patients. In ICU admission and COVID-19 death by age, the higher the age, the higher the ICU admission and COVID-19 death rate. However, the ICU admission rates for patients aged 0–9 years (12.3%) were higher than those for patients aged 40–49 years (10.2%). The ICU admission rate of COVID-19 patients was highest in the other areas (29.2%), while the COVID-19 mortality rate was higher in Gyeongsangbuk-do (5.8%). Patients with medical benefits (17.7%) had the highest ICU admission rates by health insurance premium level, and these rates increased as the health insurance premium level increased in general. The COVID-19 mortality rates showed a similar trend to the ICU admission rates. Higher CCI scores were associated with higher ICU admission and COVID-19 mortality rates.

### 3.2. TB and COVID-19 Infection, ICU Admission, and Mortality

TB was not related to COVID-19 infection (*p* = 0.88, Table 1), but it was related to ICU admission after COVID-19 infection (*p* < 0.05, Table 1). In addition, TB was not related with the mortality after COVID-19 infection (*p* < 0.05, Table 1).

### 3.3. Logistic Regression Analysis for SARS-CoV-2 Infection

Multivariate logistic regression analysis was performed in order to evaluate the impact of social factors on COVID-19 severity (Table 2). SARS-CoV-2 infection was used as the outcome variable in two logistic regression models, and the study revealed a link between SARS-CoV-2 infection and the most independent variables in the logistic regression model. Health insurance premiums, CCI scores, and tuberculosis were adjusted in multivariate model 1, and health insurance premiums, CCI scores, disability, and tuberculosis were adjusted in multivariate model 2. First, an analysis of the health insurance premium level of the COVID-19 patients showed that multivariate model 1 and multivariate model 2 were higher in patients with medical benefits. The higher the CCI score, the higher the risk of SARS-CoV-2 infection; this was statistically significant in models 1 and 2. However, statistical significance was not observed in patients with a CCI score of 1 compared with those with a CCI score of 0. The diagnosis of TB did not affect SARS-CoV-2 infection in either multivariate model 1 or 2.

### 3.4. Logistic Regression Analysis for ICU Admission in COVID-19 Patients

Multivariate logistic regression analysis was performed in order to evaluate the impact of social factors on COVID-19 severity (Table 3). Two logistic regression models were created with ICU admission as the outcome variables, and the analysis showed an association between ICU admission and the most independent variables in the logistic regression model. Multivariate model 1 was adjusted for sex, age, region of residence, health insurance premium, CCI score, and tuberculosis, and multivariate model 2 was adjusted for sex, age, region of residence, health insurance premium, CCI score, disability, and tuberculosis. First, male patients had a higher probability of ICU admission. Patients under the age of 80 were at a lower probability of being admitted to the ICU. Patients aged 0–59 years had the lowest severity. By region, the risk of ICU admission was highest in the ‘Others’. However, statistical significance was not observed in patients who resided in Gyeonggi-do. The analysis of the health insurance premium level of ICU admission showed that multivariate models 1 and 2 were higher in patients with medical benefits. The higher the CCI score, the higher the risk of ICU admission, which was statistically significant. However, statistical significance was not observed in patients with a CCI score of 1 or 3+. The risk of ICU admission was higher in patients with tuberculosis according to multivariate model 1 (OR, 4.087; 95% CI, 1.223–13.663) and 2 (OR, 4.278; 95% CI, 1.272–14.386).

### 3.5. Logistic Regression Analysis for COVID-19 Death in COVID-19 Patients

Multivariate logistic regression analysis was performed in order to evaluate the impact of social factors on the death of COVID-19 patients (Table 4). Two logistic regression models were created with COVID-19 death as the outcome variables, and the analysis showed an association between COVID-19 death and the most independent variables in the logistic regression model. Sex, age, region of residence, health insurance premium, CCI score, disability, and tuberculosis were all adjusted in multivariate model 1, and sex, age, region of residence, health insurance premium, CCI score, disability, and TB were all adjusted in multivariate model 2. First, the risk of COVID-19 death was higher in male patients. Younger age groups were at a lower risk of COVID-19 death than patients over 80 years of age. Patients aged 0–59 years had the lowest severity. By region, the risk of COVID-19 death was highest in Gyeonggi-do. However, statistical significance was observed in patients who resided in Seoul and Gyeonggi-do. The analysis of the health insurance premium level of COVID-19 deaths showed that multivariate models 1 and 2 were higher in patients with medical benefits. The risk of COVID-19 death was the highest, with a CCI score of 1. 

## 4. Discussion

In this study, we analyzed 129,128 people in the NHIS-COVID DB, including COVID-19 patients and controls, between 1 January and 30 May 2020, during the early stage of the COVID-19 epidemic in South Korea, when social distancing was not reinforced. Infection, ICU admission, and mortality due to COVID-19 increased in socioeconomically and physically vulnerable groups. However, the relationship between TB and SARS-CoV-2 infection and mortality was not evident, possibly owing to the small number of TB cases.

In the early phase of the epidemic, before the official announcement of social distancing from the Korean government, morbidity and death in the vulnerable group increased. Given that underserved populations were disproportionately affected by higher rates of both acute and chronic illness, received lower quality care, and experienced worse health-related outcomes [11], the patterns of SARS-CoV-2 infection seem to be similar in the early phase of the epidemic, when hospital beds were overwhelmed. Therefore, attention must be paid to the social needs of disadvantaged subpopulations that are not unnoticed during the COVID-19 pandemic [12]. 

Similarly to other reports, our study showed a higher rate of ICU admission and mortality in men and older adults [13]. In addition, the severity of underlying disease was related to SARS-CoV-2 infection and mortality, similarly to when the Charlson comorbidity index (CCI) was used as the comorbidity index [14], which means that higher mean CCI scores were associated with mortality [15]. However, the ICU admission rate was not related to the CCI score because the priority of ICU admission was not guided based on the COVID-19 severity score in the initial COVID-19 surge [15,16]. However, other comorbid conditions, such as obesity and peripheral vascular disease, must be investigated in order to determine their relationship with and influence on COVID-19 mortality. 

TB was not related to COVID-19 infection, but was related to ICU admission after COVID-19 infection—excluding mortality from COVID-19—in our small dataset. Patients with COVID-19 who had recovered from TB seem to be devastated because of residual compromised lung function from TB sequelae [9]. Therefore, destructive changes in TB could contribute to respiratory failure with COPD symptoms, resulting in ICU admission [17]. In addition, as reported in the Philippines, time-to-recovery was reported to be significantly longer in COVID-19 patients with TB than in those without TB [18].

In our study, the included number of TB patients was only 216 patients for 5 months, which is a relatively low number compared with the average of 9925 patients for 5 months in 2019 [19].

The reduced reporting rate of TB could have resulted from decreased transmission associated with physical distancing and the increased use of face masks. In addition, the re-allocation of vacant human resources for TB contact investigation, the difficult accessibility of hospital clinics owing to COVID-19 pre-screening, and prioritized negative pressure units for admitted COVID-19 patients are influential reasons for the reduced TB reporting.

This study has several limitations. First, this study was performed based on the National Health Insurance System DB, without more accurate or precise clinical information in the early epidemic stage. Therefore, a more precise investigation with recent data in all regions in the midst of COVID-19 is warranted. Second, the reported TB numbers in this study are small; therefore, greater numbers of TB cases in the national TB control program DB should be joined with the COVID-19 DB in order to clarify the relationship between TB and SARS-CoV-2 infection. In addition, microbiologically confirmed TB cases excluding false positive diagnosis from imaging-only studies must be selected in order to decide the definite biological relationships between TB and COVID-19 infection. Lastly, because these data were taken before the settlement of triage for the intensive care unit capacity based on clinical severity score (Central Disease Control Headquarters, 2020) [20], an updated recent national analysis under the control of a governmental administrative order for ICU arrangement should be performed.

However, we concluded that—based on NHIS data in the early epidemic—SARS-CoV-2 infection, ICU admission, and mortality from COVID-19 were increased in socioeconomically and physically vulnerable groups. However, the relationship between TB and SARS-CoV-2 infection and mortality must be elucidated with a greater number of TB cases, revealing the possible underestimation of TB reporting in the COVID-19 pandemic era.

## 5. Conclusions

SARS-CoV-2 infection, ICU hospitalization, and COVID-19 mortality increased in socioeconomically and physically vulnerable populations during the early pandemic. However, because of suspected under-reporting of TB cases and the small number of TB patients, the link between tuberculosis, COVID-19, and death was not conclusive. With a larger number of TB cases, the connection between TB and SARS-CoV-2 infection and mortality can be better understood, exposing the potential underreporting of TB in the COVID-19 pandemic timeframe.

## Figures and Tables

**Table 1:** Baseline characteristics.

		SARS-CoV-2 Infection					ICU Admission					COVID-19 Death				
		No		Yes		*p*-Value	No		Yes		*p*-Value	No		Yes		*p*-Value
		n	%	n	%		n	%	n	%		n	%	n	%	
Sex	Male	48,539	93.7	3244	6.3	1	2729	84.1	515	15.9	<0.0001 *	3108	95.8	136	4.2	<0.0001 *
	Female	72,509	93.7	4836	6.3		4331	89.6	505	10.4		4724	97.7	112	2.3	
Age, yr	0–59	87,733	93.7	5856	6.3	1	5314	90.7	542	9.3	<0.0001 *	5836	99.7	20	0.3	<0.0001 *
	60–69	17,985	93.7	1200	6.3		995	82.9	205	17.1		1162	96.8	38	3.2	
	70–79	9255	93.7	618	6.3		467	75.6	151	24.4		552	89.3	66	10.7	
	80+	6075	93.7	406	6.3		284	70.0	122	30.0		282	69.5	124	30.5	
Region of residence	Seoul	8160	93.7	549	6.3	1	446	81.2	103	18.8	<0.0001 *	545	99.3	4	0.7	<0.0001 *
	Daegu	78,960	93.8	5264	6.3		4835	91.9	429	8.1		5112	97.1	152	2.9	
	Gyeonggi-do	6764	93.7	455	6.3		336	73.8	119	26.2		438	96.3	17	3.7	
	Gyeongsangbuk-do	14,340	93.8	956	6.3		837	87.6	119	12.4		901	94.2	55	5.8	
	Others	12,824	93.7	856	6.3		606	70.8	250	29.2		836	97.7	20	2.3	
Health insurance premium	Medical aid	6560	89.1	803	10.9	<0.0001 *	661	82.3	142	17.7	<0.0001 *	754	93.9	49	6.1	<0.0001 *
	1st quintile (lowest)	22,807	93.4	1602	6.6		1433	89.5	169	10.5		1565	97.7	37	2.3	
	2nd quintile	18,039	94.2	1102	5.8		994	90.2	108	9.8		1082	98.2	20	1.8	
	3rd quintile	20,518	94.0	1312	6.0		1157	88.2	155	11.8		1280	97.6	32	2.4	
	4th quintile	22,941	94.5	1348	5.5		1188	88.1	160	11.9		1310	97.2	38	2.8	
	5th quintile (highest)	30,183	94.0	1913	6.0		1627	85.0	286	15.0		1841	96.2	72	3.8	
CCI Score	0	99,311	93.9	6469	6.1	<0.0001 *	5742	88.8	727	11.2	<0.0001 *	6381	98.6	88	1.4	<0.0001 *
	1	10,603	93.7	710	6.3		606	85.4	104	14.6		666	93.8	44	6.2	
	2	8573	92.7	680	7.3		545	80.1	135	19.9		604	88.8	76	11.2	
	3+	2561	92.1	221	7.9		167	75.6	54	24.4		181	81.9	40	18.1	
Tuberculosis	No	120,845	93.7	8067	6.3	0.8847	7053	87.4	1014	12.6	0.0003 *	7821	97.0	246	3.0	0.01 *
	Yes	203	94.0	13	6.0		7	53.8	6	46.2		11	84.6	2	15.4	

Abbreviations: ICU, intensive care unit; COVID-19, coronavirus disease 2019; CCI, Charlson comorbidity index. * *p* < 0.05, *p* value for χ^2^ test.

**Table 2:** Odds ratio for the risk of SARS-CoV-2 infection.

		Multivariate Model 1				Multivariate Model 2			
		Adjusted OR	95% CI		*p*-Value	Adjusted OR	95% CI		*p*-Value
			LB	UB			LB	UB	
Health insurance premium	Medical aid	1 (ref)	1 (ref)	1 (ref)	1 (ref)	1 (ref)	1 (ref)	1 (ref)	1 (ref)
	1st quintile (lowest)	0.584	0.534	0.639	<0.0001 *	0.604	0.552	0.661	<0.0001 *
	2nd quintile	0.509	0.463	0.56	<0.0001 *	0.528	0.479	0.582	<0.0001 *
	3rd quintile	0.532	0.485	0.584	<0.0001 *	0.551	0.502	0.605	<0.0001 *
	4rd quintile	0.488	0.445	0.535	<0.0001 *	0.505	0.46	0.555	<0.0001 *
	5th quintile (highest)	0.524	0.481	0.572	<0.0001 *	0.542	0.496	0.592	<0.0001 *
CCI Score	0	1 (ref)	1 (ref)	1 (ref)	1 (ref)	1 (ref)	1 (ref)	1 (ref)	1 (ref)
	1	1.005	0.927	1.089	0.9108	0.992	0.915	1.075	0.8394
	2	1.173	1.081	1.274	0.0001 *	1.152	1.06	1.251	0.0008 *
	3+	1.23	1.069	1.416	0.0039 *	1.19	1.033	1.371	0.0159 *
Tuberculosis	No	1 (ref)	1 (ref)	1 (ref)	1 (ref)	1 (ref)	1 (ref)	1 (ref)	1 (ref)
	Yes	0.895	0.51	1.571	0.6993	0.884	0.504	1.553	0.6689

The *p*-values were obtained by multivariate logistic analysis. * Significant difference noted (*p* < 0.05). Abbreviations: ICU, intensive care unit; OR, odds ratio; CI, confidence interval; CCI, Charlson comorbidity index.

**Table 3:** Odds ratio for the risk of ICU admission.

		Multivariate Model 1				Multivariate Model 2			
		Adjusted OR	95% CI		*p*-Value	Adjusted OR	95% CI		*p*-Value
			LB	UB			LB	UB	
Sex	Male	1 (ref)	1 (ref)	1 (ref)	1 (ref)	1 (ref)	1 (ref)	1 (ref)	1 (ref)
	Female	0.656	0.57	0.754	<0.0001 *	0.672	0.584	0.774	<0.0001 *
Age, yr	0–59	1 (ref)	1 (ref)	1 (ref)	1 (ref)	1 (ref)	1 (ref)	1 (ref)	1 (ref)
	60–69	2.447	2.025	2.956	<0.0001 *	2.338	1.933	2.829	<0.0001 *
	70–79	3.957	3.158	4.958	<0.0001 *	3.685	2.933	4.629	<0.0001 *
	80+	4.897	3.731	6.427	<0.0001 *	4.437	3.363	5.855	<0.0001 *
Region of residence	Seoul	1 (ref)	1 (ref)	1 (ref)	1 (ref)	1 (ref)	1 (ref)	1 (ref)	1 (ref)
	Daegu	0.276	0.215	0.355	<0.0001 *	0.271	0.211	0.348	<0.0001 *
	Gyeonggi-do	1.346	0.986	1.837	0.0613	1.32	0.967	1.802	0.0807
	Gyeongsangbuk-do	0.353	0.259	0.48	<0.0001 *	0.332	0.243	0.452	<0.0001 *
	Others	1.67	1.277	2.184	0.0002 *	1.651	1.263	2.159	0.0002 *
Health insurance premium	Medical aid	1 (ref)	1 (ref)	1 (ref)	1 (ref)	1 (ref)	1 (ref)	1 (ref)	1 (ref)
	1st quintile (lowest)	0.594	0.459	0.769	<0.0001 *	0.67	0.514	0.873	0.003 *
	2nd quintile	0.555	0.417	0.739	<0.0001 *	0.639	0.476	0.859	0.003 *
	3nd quintile	0.639	0.49	0.832	0.0009 *	0.735	0.559	0.965	0.0269 *
	4nd quintile	0.521	0.4	0.679	<0.0001 *	0.598	0.455	0.785	0.0002 *
	5th quintile (highest)	0.589	0.463	0.75	<0.0001 *	0.676	0.527	0.869	0.0022 *
CCI Score	0	1 (ref)	1 (ref)	1 (ref)	1 (ref)	1 (ref)	1 (ref)	1 (ref)	1 (ref)
	1	1.129	0.888	1.436	0.3211	1.099	0.864	1.398	0.4429
	2	1.383	1.098	1.742	0.0058 *	1.362	1.081	1.718	0.0089 *
	3+	1.342	0.938	1.919	0.1072	1.302	0.907	1.868	0.1525
Tuberculosis	No	1 (ref)	1 (ref)	1 (ref)	1 (ref)	1 (ref)	1 (ref)	1 (ref)	1 (ref)
	Yes	4.087	1.223	13.663	0.0222 *	4.278	1.272	14.386	0.0188 *

The *p*-values were obtained by multivariate logistic analysis. * Significant difference noted (*p* < 0.05). Abbreviations: ICU, intensive care unit; OR, odds ratio; CI, confidence interval; CCI, Charlson comorbidity index. Multivariate model 1 was adjusted for sex, age, region of residence, health insurance premium, CCI score, and tuberculosis, and multivariate model 2 was adjusted for sex, age, region of residence, health insurance premium, CCI score, disability, and tuberculosis.

**Table 4:** Odds ratio for the risk of COVID-19 death.

		Multivariate Model 1				Multivariate Model 2			
		Adjusted OR	95% CI		*p*-Value	Adjusted OR	95% CI		*p*-Value
			LB	UB			LB	UB	
Sex	Male	1 (ref)	1 (ref)	1 (ref)	1 (ref)	1 (ref)	1 (ref)	1 (ref)	1 (ref)
	Female	0.403	0.3	0.54	<0.0001 *	0.412	0.307	0.553	<0.0001 *
Age, yr	0–59	1 (ref)	1 (ref)	1 (ref)	1 (ref)	1 (ref)	1 (ref)	1 (ref)	1 (ref)
	60–69	7.154	4.104	12.472	<0.0001 *	6.83	3.91	11.933	<0.0001 *
	70–79	25.366	15.027	42.819	<0.0001 *	24.083	14.237	40.738	<0.0001 *
	80+	89.827	53.219	151.615	<0.0001 *	83.672	49.39	141.75	<0.0001 *
Region of residence	Seoul	1 (ref)	1 (ref)	1 (ref)	1 (ref)	1 (ref)	1 (ref)	1 (ref)	1 (ref)
	Daegu	2.36	0.792	7.033	0.1232	2.255	0.759	6.698	0.1434
	Gyeonggi-do	3.807	1.133	12.798	0.0307 *	3.54	1.054	11.887	0.0408 *
	Gyeongsangbuk-do	2.791	0.907	8.589	0.0734	2.627	0.856	8.062	0.0914
	Others	2.662	0.815	8.699	0.105	2.498	0.765	8.156	0.1296
Health insurance premium	Medical aid	1 (ref)	1 (ref)	1 (ref)	1 (ref)	1 (ref)	1 (ref)	1 (ref)	1 (ref)
	1st quintile (lowest)	0.476	0.29	0.782	0.0034*	0.51	0.31	0.842	0.0084 *
	2nd quintile	0.54	0.3	0.974	0.0405 *	0.601	0.331	1.092	0.095
	3nd quintile	0.691	0.414	1.154	0.158	0.773	0.459	1.301	0.332
	4nd quintile	0.496	0.304	0.808	0.0049 *	0.547	0.333	0.898	0.017 *
	5th quintile (highest)	0.507	0.329	0.782	0.0021 *	0.556	0.358	0.863	0.0089 *
CCI Score	0	1 (ref)	1 (ref)	1 (ref)	1 (ref)	1 (ref)	1 (ref)	1 (ref)	1 (ref)
	1	2.82	1.871	4.251	<0.0001 *	2.744	1.82	4.139	<0.0001 *
	2	2.67	1.856	3.84	<0.0001 *	2.618	1.818	3.771	<0.0001 *
	3+	2.395	1.504	3.813	0.0002 *	2.314	1.449	3.694	0.0004 *
Tuberculosis	No	1 (ref)	1 (ref)	1 (ref)	1 (ref)	1 (ref)	1 (ref)	1 (ref)	1 (ref)
	Yes	2.505	0.414	15.165	0.3175	2.588	0.415	16.137	0.3086

The *p*-values were obtained by multivariate logistic analysis. * Significant difference noted (*p* < 0.05). Abbreviations: ICU, intensive care unit; OR, odds ratio; CI, confidence interval; CCI, Charlson comorbidity index. Multivariate model 1 was adjusted for sex, age, region of residence, health insurance premium, CCI score, and tuberculosis, and multivariate model 2 was adjusted for sex, age, region of residence, health insurance premium, CCI score, disability, and tuberculosis.

## Data Availability

The datasets generated and analyzed during the current study are not publicly available as the National Health Insurance Service (NHIS) kept the datasets for analysis within its closed network to prevent the leak of personal information.

## References

[1] World Health Organization (2021). Weekly Operational Update on COVID-19.

[2] World Health Organization Rapid Communication on Forthcoming Changes to the Programmatic Management of Tuberculosis Preventive Treatment. http://apps.who.int/bookorders..

[3] Lim W.S., Liang C.K., Assantachai P., Auyeung T.W., Kang L., Lee W.J., Lim J., Sugimoto K., Akishita M., Chia S. (2020). COVID-19 and older people in Asia: Asian Working Group for Sarcopenia calls to actions. Geriatr. Gerontol. Int..

[4] Courtenay K., Perera B. (2020). Courtenay and Perera, COVID-19 and people with intellectual disability: Impacts of a pandemic. Ir. J. Psychol. Med..

[5] Yang J., Zheng Y., Gou X., Pu K., Chen Z., Guo Q., Ji R., Wang H., Wang Y., Zhou Y. (2020). Prevalence of comorbidities and its effects in patients infected with SARS-CoV-2: A systematic review and meta-analysis. Int. J. Infect. Dis..

[6] Ejaz H., Alsrhani A., Zafar A., Javed H., Junaid K., Abdalla A.E., Abosalif K.O.A., Ahmed Z., Younas S. (2020). COVID-19 and comorbidities: Deleterious impact on infected patients. J. Infect. Public Health.

[7] Chen Y., Wang Y., Fleming J., Yu Y., Gu Y., Liu C., Fan L., Wang X., Cheng M., Bi L. (2020). Active or latent tuberculosis increases susceptibility to COVID-19 and disease severity. MedRxiv.

[8] Korea Centers for Disease Control and Prevention 2020 Annual Report on the Notified Tuberculosis in Korea. https://www.kdca.go.kr/board/board.es?mid=a20602010000&bid=0034&act=view&list_no=712904.

[9] Udwadia Z.F., Vora A., Tripathi A.R., Malu K.N., Lange C., Raju R.S. (2020). COVID-19 -Tuberculosis interactions: When dark forces collide. Indian J. Tuberc..

[10] Quan H., Li B., Couris C.M., Fushimi K., Graham P., Hider P., Januel J.M., Sundararajan V. (2011). Updating and validating the Charlson comorbidity index and score for risk adjustment in hospital discharge abstracts using data from 6 countries. Am. J. Epidemiol..

[11] Grief S.N., Miller J.P. (2017). Infectious Disease Issues in Underserved Populations. Prim. Care Clin. Off. Pr..

[12] Quinn S.C., Kumar S. (2014). Health Inequalities and Infectious Disease Epidemics: A Challenge for Global Health Security. Biosecurity Bioterrorism Biodefense Strategy Pract. Sci..

[13] Cannistraci C.V., Valsecchi M.G., Capua I. (2021). Age-sex population adjusted analysis of disease severity in epidemics as a tool to devise public health policies for COVID-19. Sci. Rep..

[14] Huang Y.-Q., Gou R., Diao Y.-S., Yin Q.-H., Fan W.-X., Liang Y.-P., Chen Y., Wu M., Zang L., Li L. (2014). Charlson comorbidity index helps predict the risk of mortality for patients with type 2 diabetic nephropathy. J. Zhejiang Univ. Sci. B.

[15] Kuswardhani R.T., Henrina J., Pranata R., Lim M.A., Lawrensia S., Suastika K. (2020). Charlson comorbidity index and a composite of poor outcomes in COVID-19 patients: A systematic review and meta-analysis. Diabetes Metab. Syndr. Clin. Res. Rev..

[16] Lee S.H., Park S.Y., Seon J.Y., Jeon W.H., Nam S.I., Park J.H., Park J.S., Kim H.Y., Thakkar N., Selvaraj P. (2020). Intensive Care Unit Capacity and Its Associated Risk Factors During the COVID-19 Surge in the Republic of Korea: Analysis Using Nationwide Health Claims Data. Risk Manag. Healthc. Policy..

[17] Chakrabarti B., Calverley P.M., Davies P.D. (2007). Tuberculosis and its incidence, special nature, and relationship with chronic obstructive pulmonary disease. Int. J. Chron. Obstruct. Pulmon. Dis..

[18] Sy K.T.L., Haw N.J.L., Uy J. (2020). Previous and active tuberculosis increases risk of death and prolongs recovery in patients with COVID-19. Infect. Dis..

[19] Korea Disease Control and Prevention Agency (2019). 2019 Annual Report on the Notified Tuberculosis in Korea. https://nih.go.kr/board/board.es?mid=a40303030000&bid=0034&act=view&list_no=366715.

[20] Central Disease Control Headquarters (2020). 2020 Coronavirus Infectious Disease-19 Response Guidelines (7th Edition). https://www.kdca.go.kr/board/board.es?mid=a20507020000&bid=0019.

